# Limb dominance for fifth metatarsal fracture in football players is position-specific

**DOI:** 10.1186/1757-1146-7-S1-A86

**Published:** 2014-04-08

**Authors:** Tomoya Ueda, Hiroaki Hobara, Yoshiyuki Kobayashi, Masaaki Mochimaru, Hiroshi Mizoguchi

**Affiliations:** 1 National Institute of Advanced Industrial Science and Technology, Tokyo, 135-0064, Japan; 2 Tokyo University of Science, Chiba, 278-8510, Japan

## 

**Figure 1:**
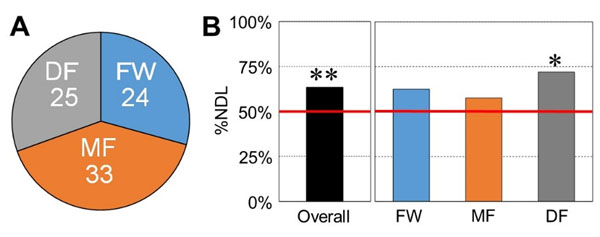
A: Incidence of fifth metatarsal fracture in three groups. B: Percentage of non-dominant limb (%NDL) for fifth metatarsal fracture in each group.

Fifth metatarsal fractures (5MtF) are one of the most common traumatic foot injuries in football player [1,2]. A previous study demonstrated that the 5MtF in football players occur frequently in their non-dominant limb [2]. Since different playing positions requires different physical demands in match-play [3], the aim of this study was to examine the hypothesis that the limb dominance for 5MtF is position-specific. Using a publicly-available injured reserve list in Japan professional football league (J-League) during 2008-2013 seasons, we collected a total of 82 cases of 5MtF. Positions (forward players: FW, midfielders: MF, and defenders: DF) and limb dominance in each player was also identified by officially-released profile in their team. To test whether the percentage of limb dominance of 5MtF differed from chance, we used a binomial test to compare reported incidence of 5MtF in non-dominant limb out of all cases to the theoretical probability of 50%. In the present study, 24 (29.3%), 33 (40.2%) and 25 (30.5%) cases of 82 cases were classified into FW, MF and DF, respectively (Figure 1-A). There were no significant differences in the incidence of 5MtF among three groups (*p*=0.41, Chi-square test). Overall, the 5MtF tended to be occurred in non-dominant limb (Figure 1-B; *p*<0.01). However, as shown in Figure 1-B, the trend was more pronounced in DF (*p*<0.01), and not in FW (*p*=0.15) and MF (*p*=0.24). These results suggest that limb dominance for5MtF is position-specific in football players.
